# Can Functional Micro-organisms Associated with Pumpkin Sizes Be Sought Out from the Soil?—A Comparison of Soil Microbial Community Structures in Rhizospheres between Giant- and Small-Sized Pumpkin Varieties

**DOI:** 10.3390/plants13162258

**Published:** 2024-08-14

**Authors:** Yu Zhu, Xinyan Zhou, Jiaoming Li, Junqian Feng, Ziyue Huang, Baoling Chen, Wenjun Liu, Shangdong Yang

**Affiliations:** 1 Guangxi Key Laboratory of Agro-Environment and Agro-Products Safety, National Demonstration Center for Experimental Plant Science Education, Guangxi Agricultural College, Guangxi University, 100 University Road, Nanning 530004, China; 15660853993@163.com (Y.Z.); zhouxinyan0923@163.com (X.Z.); 13877153675@163.com (J.L.); f1026@126.com (J.F.); 13978350152@163.com (Z.H.); 2 Vegetable Research Institute, Guangxi Academy of Agricultural Sciences, Nanning 530007, China; chenbl1990721@163.com (B.C.); liuwenjun@gxaas.net (W.L.)

**Keywords:** pumpkin, soil microbial community structure, rhizosphere, yield, functional microorganisms

## Abstract

To elucidate the biological mechanisms driving the growth of various pumpkin varieties to different sizes under identical management conditions while in the same field, the soil microbial community structures in the rhizospheres of giant-pumpkin (GP) and small-pumpkin (SP) varieties were analyzed. The results revealed that a significantly higher abundance of bacterial communities could be detected in the rhizospheres of the giant pumpkin varieties, such as Gemmatimonadota, *norank__f__norank__o_Gaiellales*, *norank__f__Gemmatimonadaceae*, *Bryobacter*, *Sphingomonas*, *norank__f__JG30-KF-AS9*, and *norank__f__norank__o___Elsterales*, than in those of the small-sized pumpkins. Additionally, *norank_f__norank_o__Elsterale*, *Ellin6067*, *norank_f__67-14*, and *Chujaibacter* were unique dominant soil bacteria genera in the rhizospheres of the giant pumpkins. By contrast, *Arthrobacter*, *norank_f__Roseiflexaceae*, *unclassified_f__Rhizobiaceae*, *Allorhizobium-Neorhizobium-Pararhizobium-Rhizobium*, *Nocardioides*, *Mycobacterium, norank_f__norank_o__Vicinamibacterales*, and *Burkholderia-Caballeronia-Paraburkholderia* were the unique dominant soil bacterial genera in the rhizospheres of the small pumpkins. Moreover, at the fungal genus level, *unclassified_c__Chytridiomycetes, Podosphaera*, and *Colletotrichum* presented significant differences between the giant-pumpkin (GP) and small-pumpkin (SP) rhizospheres. In addition, *unclassified__p__Rozellomycota*, *unclassified__c__Chytridiomycetes*, *Penicillium*, and *unclassified__f__Chaetomiaceae* were unique dominant soil fungal genera in the rhizospheres of the giant pumpkins (GPs). By contrast, *Podosphaera*, *Colletotrichum*, *unclassified__f__Plectosphaerellaceae*, *unclassified__o_Boletales*, *Scytalidium*, *unclassified__p__Rozellomycota*, and *unclassified__o_Agaricales* were the unique dominant soil fungal genera in the rhizospheres of the small pumpkins (SPs). PICRUSt and FUNGuild functional prediction analyses revealed that the giant-pumpkin rhizosphere microbial community had significantly increased translation, ribosomal structure and biogenesis, nucleotide transport and metabolism, defense mechanisms, replication, recombination and repair, wood saprotroph, and undefined saprotroph levels. The above results suggest that the soil microbial compositions differed between the rhizospheres of the giant- (GP) and small-pumpkin (SP) varieties, even though the plants were grown in the same field under identical management conditions. Meanwhile, bacterial genera such as *norank_f__norank_o__Elsterale*, *Ellin6067*, *norank_f__67-14*, and *Chujaibacter*, in addition to fungal genera such as *unclassified__p__Rozellomycota*, *unclassified__c__Chytridiomycetes*, *Penicillium*, and *unclassified__f__Chaetomiaceae*, can be speculated as potential soil functional micro-organisms associated with improved pumpkin size.

## 1. Introduction

The pumpkin (*Cucurbita moschata* Duch.) is an annual herbaceous plant of the Cucurbitaceae family, which originated from Mexico in Central America [1,2]. Pumpkin was introduced into China during the Ming Dynasty, and it is now cultivated all over the country [3]. At present, the main planted pumpkin species are the Chinese Pumpkin (*Cucurbita moschata* Duch.), Indian Pumpkin (*Cucurbita maxima* Duch ex lam.), Black Seed Pumpkin (*Cucurbita fieifolia.*), and Mexican Pumpkin (*Cucurbita mixta.*), among others [4]. Additionally, according to the pumpkin fruit morphology, pumpkins can be divided into giant and small sizes. Moreover, giant- and small-sized pumpkins are mainly used for ornamental reasons and food, respectively [5,6].

Previous studies have shown that pumpkin size is affected by many factors, such as genes [7] and external environmental factors (e.g., light, water, and temperature) [8]. In particular, soil conditions are critical for the development of pumpkin size, as they are not only important for plant growth but are also important for the survival of microorganisms in nature, as soil is their main habitat [9,10]. The rhizosphere is not only where plants actively absorb nutrients from the soil but is also an important ecological location for micro-organisms [11]. As 20–40% of organic matter is transported to the roots through plant photosynthesis [12], micro-organisms gathering in the rhizosphere can obtain nutrients from the roots. In addition, micro-organisms living in the rhizosphere affect plant growth and development through the production of plant hormones, promoting the soil nutrient cycle, reducing harmful micro-organisms, and regulating plant gene expression levels, among other aspects [13,14].

For instance, while it is difficult for plants to directly absorb phosphorus from the soil, they can freely utilize it after the action of micro-organisms in the rhizosphere. Furthermore, phytohormones such as auxin, cytomin, ethylene, abscisic acid, jasmonic acid, and salicylic acid can be produced by the micro-organisms in the rhizosphere, promoting nutrient uptake and utilization by plants [15,16,17]. Disease and stress resistance can also be promoted by micro-organisms living in the rhizosphere [18]. Moreover, organic compounds, such as alcohols, esters, terpenoids, and ketones, can also be derived from micro-organisms in the rhizosphere [19,20,21].

As is well known, giant-sized pumpkins can reach tens to hundreds of kilograms, while small-sized pumpkins weigh only 0.5 kg or less. Even though giant- and small-sized pumpkins grow in the same fields and under identical management conditions, they can show significant differences in fruit size, thus posing some very interesting questions. For example, on Earth, what types of soil micro-organisms enrich rhizospheres? How are these micro-organisms associated with pumpkin size? Therefore, the soil microbial community compositions in the rhizospheres of giant- and small-sized pumpkin varieties were analyzed in this study.

## 2. Results

### 2.1. Soil Bacterial Diversity in Rhizospheres between Giant- and Small-Sized Pumpkin Varieties

As shown in Table 1, all coverage rates reached above 97%, and the data were considered reliable (Table 1). The Shannon, Simpson, Ace, and Chao1 indices of soil bacterial diversity and richness in the rhizospheres of the giant- and small-sized pumpkin varieties did not significantly differ among each other (*p* < 0.05).

### 2.2. Soil Bacterial Community Composition in the Rhizosphere between Giant- and Small-Sized Pumpkin Varieties

#### 2.2.1. Analysis of the Rhizospheric Soil Bacterial Compositions

**Figure 1:**
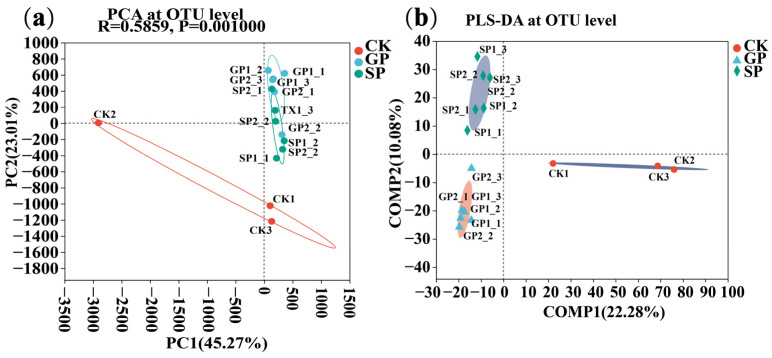
Soil bacterial compositions in the rhizospheres of giant- and small-sized pumpkin varieties: (**a**) PCoA of the rhizosphere soil bacterial communities at the OTU level; and (**b**) PLS-DA of the rhizosphere soil bacterial communities at the OTU level.

Principal coordinate analysis (PCoA) and partial least-squares discriminant analysis (PLS-DA) at the operational taxonomic unit (OTU) level were conducted to evaluate the extent of the similarity of the rhizospheric bacterial communities. The results showed that the rhizospheric bacterial communities of the GPs and SPs were significantly different from CK (*p* < 0.05). Meanwhile, it also found that the rhizospheric bacterial communities of the GPs, SPs, and CK were clustered separately. These results suggest that the soil bacterial compositions in the rhizospheres of the giant- and small-sized pumpkin varieties were significantly different (Figure 1).

**Figure 2:**
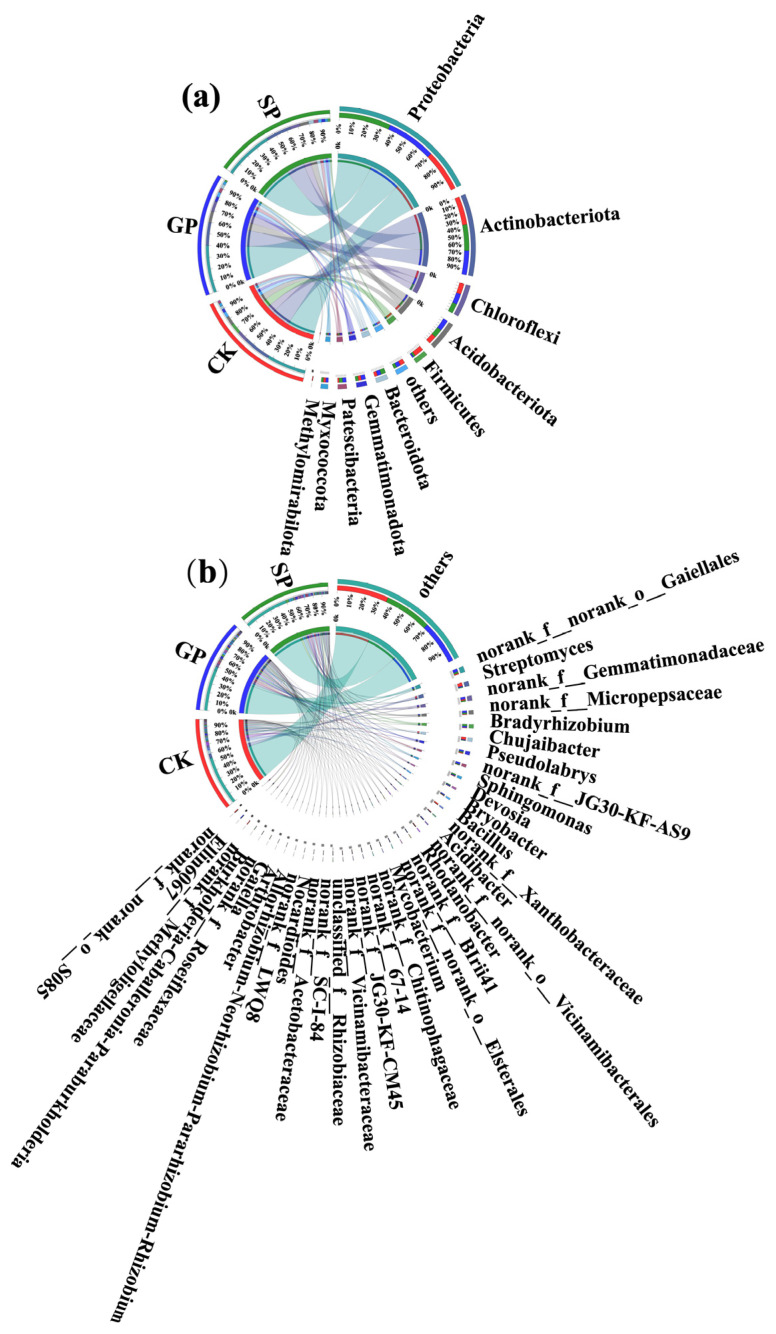
Distribution of dominant soil bacteria in the rhizospheres of giant-sized pumpkin (GP) and small-sized pumpkin (SP) varieties and background (CK) at the phylum (**a**) and genus (**b**) levels. “norank” is a taxonomic term indicating that there is no clear taxonomic information or taxonomic name at a taxonomic level.

At the phylum level, there were nine dominant soil bacterial phyla (i.e., those with proportions greater than 1%) in each of the GP, SP, and CK rhizospheres (Figure 2a). First, the proportions of dominant soil bacterial phyla in the rhizospheres of the giant-sized pumpkin (GP) varieties from high to low were Proteobacteria (40.82%), Actinobacteriota (20.07%), Acidobacteriota (9.60%), Chloroflexi (9.20%), Gemmatimonadota (4.31%), Patescibacteria (3.88%), Bacteroidota (3.87%), Myxococcota (2.78%), and Firmicutes (1.40%). By contrast, the proportions of dominant soil bacterial phyla for the small-sized pumpkin (SP) varieties from high to low were Proteobacteria (42.82%), Actinobacteriota (21.94%), Chloroflexi (8.61%), Acidobacteriota (7.79%), Bacteroidota (4.59%), Patescibacteria (3.77%), Gemmatimonadota (2.92%), Myxococcota (2.40%), and Firmicutes (1.97%). Meanwhile, the proportions of dominant soil bacterial phyla in the background (CK) sample from high to low were Proteobacteria (34.33%), Actinobacteriota (23.59%), Chloroflexi (9.43%), Firmicutes (9.19%), Acidobacteriota (7.35%), Gemmatimonadota (3.04%), Bacteroidota (2.94%), Myxococcota (2.63%), and Patescibacteria (1.49%).

The above results suggest that the proportions of Patescibacteria, Myxococcota, Acidobacteriota, Chloroflexi, and Gemmatimonadota were greater in the rhizospheres of the giant-sized pumpkin varieties than in the rhizospheres of the small-sized pumpkins. On the contrary, the proportions of Bacteroidota, Firmicutes, Bacteroidota, Firmicutes, Proteobacteria, and Actinobacteriota were lower in the rhizospheres of the giant-sized pumpkin varieties than in those of the small-sized pumpkin varieties.

**Figure 3:**
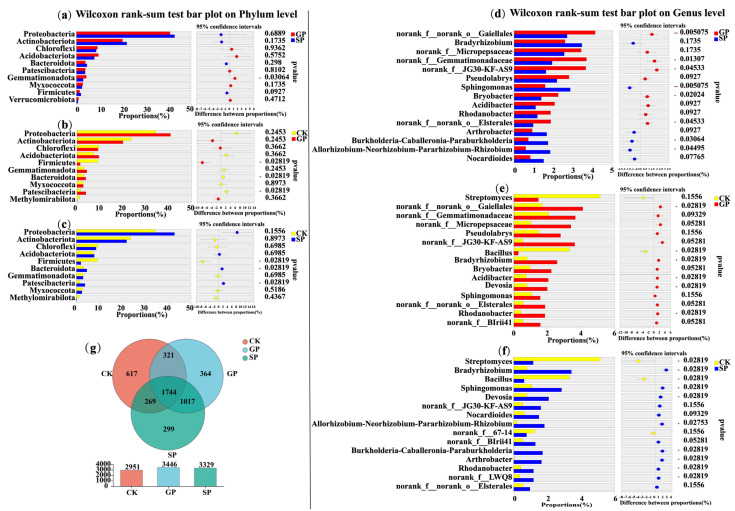
Significance analysis of soil bacteria in the rhizospheres of giant-sized pumpkin (GP) and small-sized pumpkin (SP) varieties and background (CK) at the phylum (**a**–**c**) and genus (**d**–**f**) levels, as well as Venn diagram analysis at the OTU level (**g**).

At the genus level, the numbers of dominant soil bacterial genera (proportions greater than 1%) in the GP, SP, and CK rhizospheres were 22, 26, and 19, respectively (Figure 3b). First, *norank__f__norank__o___Gaiellales* (4.11%), *norank__f__Gemmatimonadaceae* (3.66%), *norank__f__JG30-KF-AS9* (3.63%), *norank__f__Micropepsaceae* (3.40%), *Pseudolabrys* (2.78%), *Bradyrhizobium* (2.58%), *Bryobacter* (2.23%), *Acidibacter* (2.05%), *Devosia* (1.99%), *Ellin6067* (1.18%), *Norank__f__norank__o___Elsterales* (1.86%), *Rhodanobacter* (1.85%) *norank__f__Acetobacteraceae* (1.69%), *norank__f__BIrii41* (1.58%), *Sphingomonas* (1.57%), *Streptomyces* (1.46%), *norank__f__LWQ8* (1.31%), *norank__f__67-14* (1.30%), *norank__f__Xanthobacteraceae* (1.24%), *Chujaibacter* (1.19%), *norank__f__Chitinophagaceae* (1.16%), and *norank__f__SC-I-84* (1.12%) were the dominant soil bacterial genera in the rhizospheres of the giant-sized pumpkin varieties. In contrast, *Bradyrhizobium* (3.43%), *Sphingomonas* (2.85%), *norank__f__norank__o___Gaiellales* (2.68%), *norank__f__Micropepsaceae* (2.54%), *Pseudolabrys* (2.17%), *Devosia* (2.08%), *norank__f__Gemmatimonadaceae* (1.92%), *Allorhizobium-Neorhizobium-Pararhizobium-Rhizobium* (1.83%), *Burkholderia-Caballeronia-Paraburkholderia* (1.72%), *Arthrobacter* (1.65%), *norank__f__JG30-KF-AS9* (1.61%), *Nocardioides* (1.50%), *Mycobacterium* (1.46%), *Bryobacter* (1.38%), *norank__f__BIrii41* (1.29%), *norank__f__Acetobacteraceae* (1.25%), *norank__f__SC-I-84* (1.24%), *norank__f__LWQ8* (1.18%), *Rhodanobacter* (1.18%), *norank__f__Roseiflexaceae* (1.18%), *Streptomyces* (1.16%), *Acidibacter* (1.09%), *unclassified__f__Rhizobiaceae* (1.13%), *norank__f__Chitinophagaceae* (1.06%), *norank__f__ Xanthobacteraceae* (1.06%), and *norank__f__norank__o___Vicinamibacterale*s (1.05%) were the dominant soil bacterial genera in the rhizospheres of the small-sized pumpkin varieties. Meanwhile, *Streptomyces* (5.13%), *Chujaibacter* (4.51%), *Bacillus* (3.32%), *norank_f__Gemmatimonadaceae* (2.07%), *norank_f__Methyloligellaceae* (1.78%), *norank_f__norank_o___Vicinamibacterales* (1.72%), *Gaiella* (1.64%), *norank_f__Vicinamibacteraceae* (1.71%), *norank__f__norank__o___Gaiellale*s (1.69%), *norank__f__Xanthobacteraceae* (1.59%), *norank__f__JG30-KF-CM45* (1.54%), *Pseudolabrys* (1.48%), *norank__f__67-14* (1.24%), *unclassified__f__Rhizobiaceae* (1.18%), *Mycobacterium* (1.15%), *Sphingomonas* (1.08%), *norank__f__Chitinophagaceae* (1.08%), and *norank__f__norank__o__ S085* (1.07%) were the dominant soil bacterial genera in the background (CK) samples.

Based on the above results, we found that *norank_f__norank_o__Elsterales* and *Ellin6067* were unique dominant soil bacterial genera in the rhizospheres of the giant-sized pumpkin varieties. By contrast, *Allorhizobium-Neorhizobium-Pararhizobium-Rhizobium*, *Arthrobacter, norank_f__Roseiflexaceae*, *Burkholderia-Caballeronia-Paraburkholderia*, and *Nocardioides* were the unique dominant soil bacterial genera in the rhizospheres of the small-sized pumpkin varieties.

Moreover, the Wilcoxon rank-sum test was carried out to analyze the significant differences in the top 10 soil bacterial phyla in the rhizospheres of the giant- and small-sized pumpkin varieties in terms of their relative abundances (Figure 3a). We found that the abundance of Gemmatimonadota in rhizospheres was significantly different between the giant- (GP) and small-sized pumpkin (SP) varieties (*p* < 0.05). Additionally, at the genus level, the proportions of *norank_f__norank_o__Gaiellales*, *norank_f__Gemmatimonadaceae*, *norank_f__JG30-KF-AS9*, *Bryobacter*, and *norank_f__norank_o_ _Elsterales* in the rhizospheres of the giant-sized pumpkin (GP) varieties were significantly higher than in those of the small-sized pumpkin (SP) varieties. The proportions of *Sphingomonas*, *Burkholderia-Caballeronia-Paraburkholderia*, and *Allorhizobium-Neorhizobium-Pararhizobium-Rhizobium* in the rhizospheres of the giant-sized pumpkin (GP) varieties were significantly lower than in those of the small-sized pumpkin (SP) varieties (Wilcoxon rank-sum test: *, 0.01 < *p* ≤ 0.05; **, 0.001 < *p* ≤ 0.01; Figure 3d).

Furthermore, at the OTU level, the numbers of soil bacteria in the rhizospheres of the giant-sized pumpkins, small-sized pumpkins, and background were 3446, 3329, and 2951 OTUs, respectively. The numbers of special bacterial OTUs in the rhizospheres of the giant-sized pumpkins, small-sized pumpkins, and background were 364, 299, and 617 OTUs, respectively (Figure 3g). These results suggest that differently sized pumpkin varieties enriched various soil bacterial compositions in their rhizospheres. Furthermore, the giant-sized pumpkin varieties recruited more unique soil bacteria in their rhizospheres.

#### 2.2.2. LEfSe Analysis of the Bacterial Communities at the Phylum and Genus Levels

**Figure 4:**
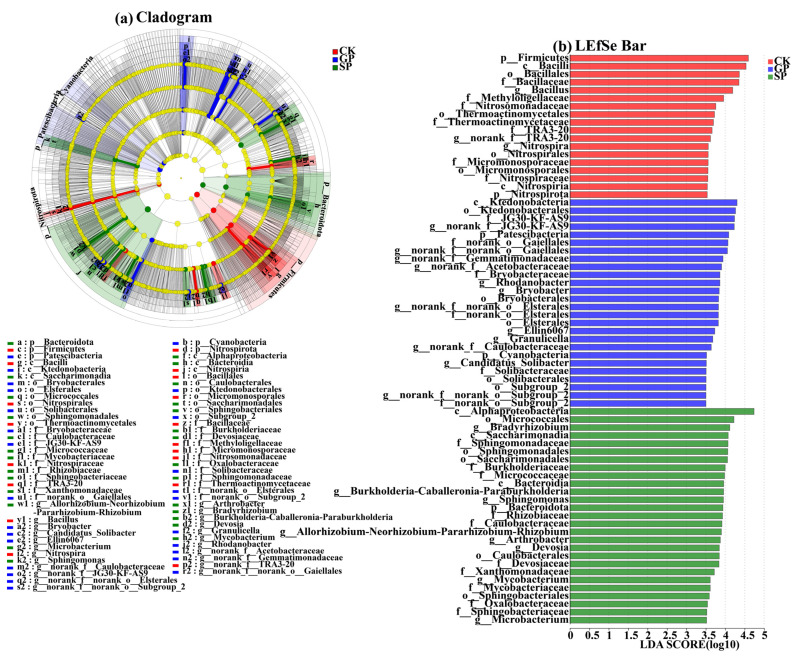
Cladogram showing the phylogenetic distribution of soil bacteria in the rhizospheres of giant-sized pumpkins (GPs), small-sized pumpkins (SPs), and the background (CK). Indicator bacteria with LDA scores of 3.5 or greater in microbial communities associated with soil from three treatments (LEfSe). Different color regions represent different constituents (blue: GP; green, SP; red: CK). Circles indicate phylogenetic level from phylum to genus. The diameter of each circle is proportional to the abundance of the group. Different prefixes indicate different levels (p: phylum; c: class, o: order; f: family; g: genus).

Linear discriminant analysis Effect Size (LEfSe) analysis was also performed on the soil bacterial communities in the rhizospheres of giant-sized pumpkins (GPs), small-sized pumpkins (SPs), and background (CK). A total of 71 rhizosphere bacterial species showed significant differences (*p* < 0.05, linear discriminant analysis [LDA] score > 3.5; Figure 4). At the phylum level, Patescibacteria and Cyanobacteria were significantly enriched in the rhizospheres of the giant-sized pumpkin (GP) varieties. In contrast, Bacteroidota were only significantly enriched in the rhizospheres of the small-sized pumpkin (SP) varieties. Meanwhile, at the genus level, *norank_f__JG30-KF-AS9*, *Candidatus_Solibacter*, *norank_f__Gemmatimonadaceae*, *norank_f__norank_o__Gaiellales*, *Granulicella*, *norank_f__Acetobacteraceae*, *Rhodanobacter*, *Bryobacter*, *norank_f__norank_o__Elsterales*, *Ellin6067*, *norank_f__Caulobacteraceae*, and *norank_f__norank_o__Subgroup_2* were significantly enriched in the rhizospheres of the giant-sized pumpkin (GP) varieties. By contrast, *Bradyrhizobium*, *Devosia*, *Burkholderia-Caballeronia-Paraburkholderia*, *Sphingomonas*, *Microbacterium*, *Mycobacterium*, *Allorhizobium-Neorhizobium-Pararhizobium-Rhizobium*, and *Arthrobacter* were enriched in the rhizospheres of small-sized pumpkin (SP) varieties.

#### 2.2.3. Functional Predictions of Soil Bacterial Community Structures

**Figure 5:**
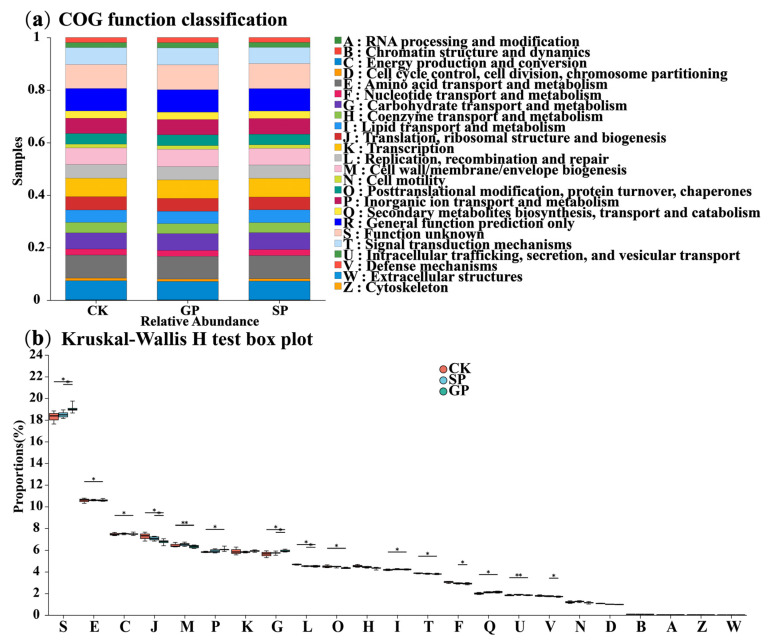
Relative abundance of PICRUSt-inferred functions (**a**) and Kruskal–Wallis rank-sum test (**b**) of soil bacteria in the rhizospheres of giant-sized pumpkins (GPs), small-sized pumpkins (SPs), and background (CK). * indicates 0.01 < *p* < 0.05; ** indicates 0 < *p* < 0.01.

Moreover, the PICRUSt function prediction method was used to predict soil bacterial functions in the rhizospheres of differently sized pumpkins (Figure 5a,b). The top 10 relative abundance ratios of the functional compositions from high to low were as follows: Function unknown > Amino acid transport and metabolism > General function prediction only > Energy production and conversion > Transcription > Carbohydrate transport and metabolism > Cell wall/membrane/envelope biogenesis > Signal transduction mechanisms > Inorganic ion transport and metabolism > Replication, recombination, and repair. Furthermore, according to the Kruskal–Wallis rank-sum test analysis, translation, ribosomal structure, and biogenesis; nucleotide transport and metabolism; defense mechanisms; and replication, recombination, and repair were significantly higher in the rhizospheres of the giant-pumpkin (GP) than in those of the small-pumpkin (SP) varieties.

### 2.3. Soil Fungal Diversity in Rhizospheres between Giant- and Small-Sized Pumpkin Varieties

The coverage rates of soil fungi in rhizospheres all reached 99%, and the data were considered reliable. As shown in Table 2, the Shannon, Simpson, Ace, and Chao1 indices of soil fungal diversity and richness in the rhizospheres of the giant-sized pumpkins (GPs), small-sized pumpkins (SPs), and background (CK) did not present significant differences (*p* < 0.05) between each other (Table 2).

### 2.4. Soil Fungal Community Composition in the Rhizosphere between Giant- and Small-Sized Pumpkin Varieties

#### 2.4.1. Analysis of the Rhizospheric Soil Fungal Compositions

**Figure 6:**
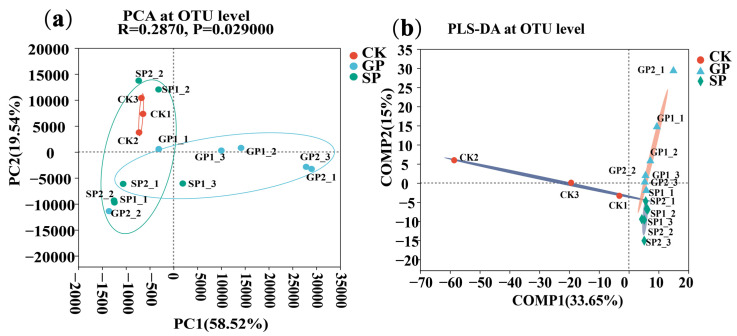
Comparison of soil fungal compositions in the rhizospheres of giant-sized pumpkins (GPs), small-sized pumpkins (SPs), and CK: (**a**) PCA analysis of soil fungal communities in rhizospheres at the OTU level; and (**b**) PLS-DA analysis of soil fungal communities in rhizospheres at the OTU level.

Based on the PCA analysis, significant differences were found between the soil fungal communities in the rhizospheres of the giant- and small-sized pumpkin varieties (Figure 6a). Meanwhile, PLS-DA analysis results also revealed that soil fungal communities in the GP, SP, and CK rhizospheres were significantly clustered. These results indicate that various soil fungal communities were recruited by the differently sized pumpkin varieties (Figure 6b).

**Figure 7:**
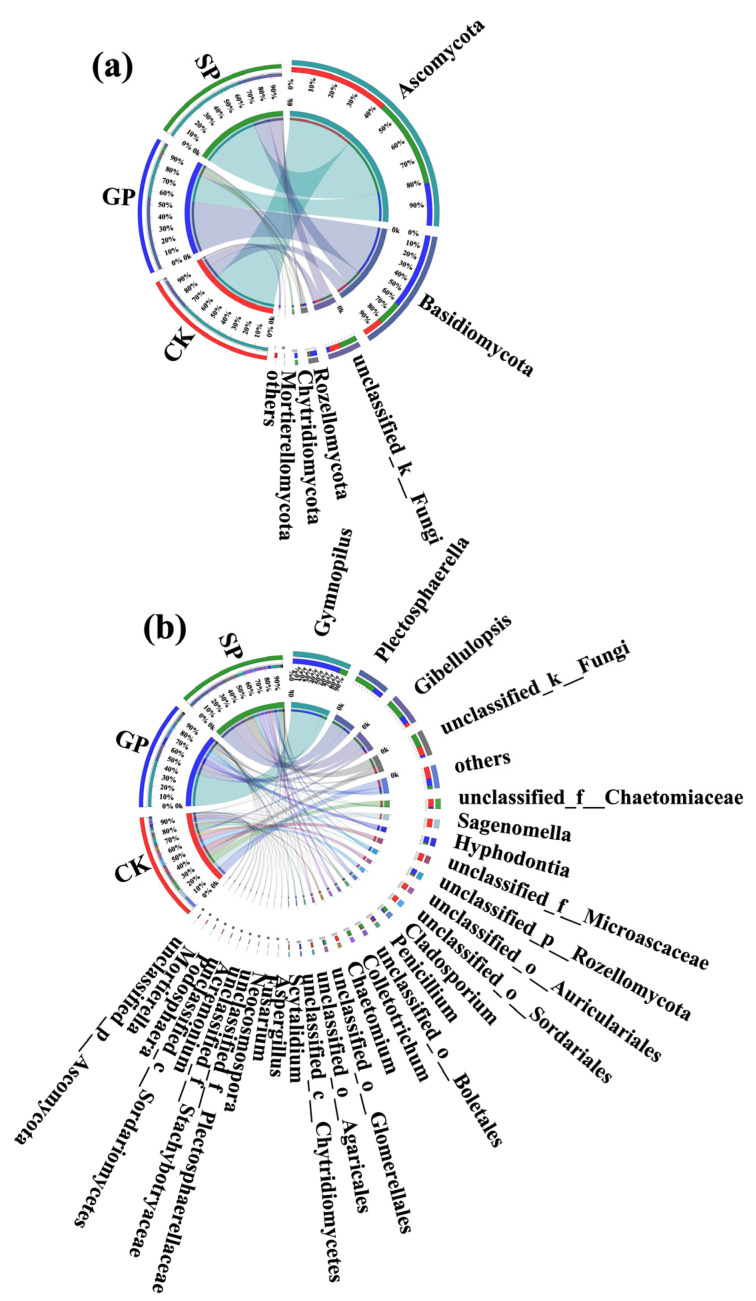
Distributions of dominant soil fungi in the rhizospheres of giant-sized pumpkins (GPs), small-sized pumpkins (SPs), and background (CK) at the phylum (**a**) and genus (**b**) levels.

At the phylum level, the numbers of dominant soil fungal phyla (proportions greater than 1%) in the rhizospheres of the GPs, SPs, and CK were 5, 4, and 5, respectively. First, Basidiomycota (56.33%), Ascomycota (31.22%), Rozellomycota (5.86%), Chytridiomycota (3.99%), and unclassified__k__Fungi (2.32%) were the dominant soil fungal phyla in the rhizospheres of the giant-sized pumpkin (GP) varieties. By contrast, Ascomycota (66.17%), Basidiomycota (17.76%), unclassified_k__Fungi (13.85%), and Rozellomycota (2.06%) were the dominant soil fungal phyla in the rhizospheres of the small-sized pumpkin (SP) varieties. Meanwhile, Ascomycota (73.45%), Basidiomycota (14.50%), unclassified_k__Fungi (9.07%), Mortierellomycota (1.85%), and Rozellomycota (1.05%) were the dominant soil fungal phyla in the background samples (CK). In comparison with the small-sized pumpkin (SP) varieties, the proportions of Basidiomycota and Rozellomycota in the rhizospheres of the giant-sized pumpkin varieties were higher. On the contrary, the proportions of Ascomycota and unclassified_k__Fungi in the rhizospheres of the giant-sized pumpkin varieties were lower than in those of the small-sized pumpkin varieties. Meanwhile, Chytridiomycota was the unique dominant soil fungal phylum in the rhizospheres of the giant-sized pumpkin varieties (Figure 7a).

Additionally, at the genus level, the numbers of the dominant soil fungal genera (proportions greater than 1%) in the rhizospheres of the GPs, SPs, and CK were 11, 14, and 21, respectively. First, *Gymnopilus* (46.10%), *Plectosphaerella* (9.42%), *Gibellulopsis* (7.46%), *Hyphodontia* (7.28%), *unclassified_p__Rozellomycota* (5.86%), *Penicillium* (3.04%), *unclassified_c__Chytridiomycetes* (3.96%), *unclassified_k__Fungi* (2.32%), *Cladosporium* (2.00%), *unclassified_o__Glomerellales* (1.40%), and *unclassified_f__Chaetomiaceae* (1.29%) were the dominant soil fungal genera in the rhizospheres of the giant-sized pumpkin (GP) varieties. In contrast, *Plectosphaerella* (21.08%), *Gibellulopsis* (19.30%), *unclassified_k__Fungi* (13.85%), *Gymnopilus* (7.82%), *Colletotrichum* (5.71%), *Cladosporium* (5.32%), *unclassified_o__Boletales* (5.24%), *Hyphodontia* (2.28%), *unclassified_o__Glomerellales* (3.95%), *unclassified_p__Rozellomycota* (2.06%), *Podosphaera* (1.86%), *unclassified_f__Plectosphaerellaceae* (1.58%), *Scytalidium* (1.27%), and *unclassified_o__ Agaricales* (1.07%) were the dominant soil fungal genera in the rhizospheres of the small-sized pumpkin (GP) varieties. Meanwhile, *Sagenomella* (9.94%), *unclassified_f__Chaetomiaceae* (9.52%), *unclassified_k__Fungi* (9.07%), *unclassified_f__Microascaceae* (8.77%), *Penicillium* (2.31%), *unclassified_o__Sordariales* (7.69%), *unclassified_o__Auriculariales* (7.65%), *Chaetomium* (5.08%), *Gibellulopsis* (3.61%), *unclassified_o__Agaricales* (3.42%), *Neocosmospora* (2.25%), *Fusarium* (2.21%), *unclassified_f__Stachybotryaceae* (2.16%), *Scytalidium* (2.02%), *Aspergillus* (2.02%), *Acremonium* (1.90%), *Mortierella* (1.74%), *unclassified_c__Sordariomycetes* (1.59%), *unclassified_p__Ascomycota* (1.22%), *unclassified_p__Rozellomycota* (1.05%)*,* and *unclassified_o__Boletales* (1.01%) were the dominant soil fungal genera in background samples (Figure 7b). The above results suggest that *unclassified_p__Rozellomycota* and *unclassified_c__Chytridiomycetes* were the unique dominant soil fungal genera in the rhizospheres of the giant-sized pumpkin (GP) varieties, while *Podosphaera*, *Colletotrichum*, and *unclassified_f__Plectosphaerellaceae* were the unique dominant soil fungal genera in the rhizospheres of the small-sized pumpkin (GP) varieties (Figure 7b).

**Figure 8:**
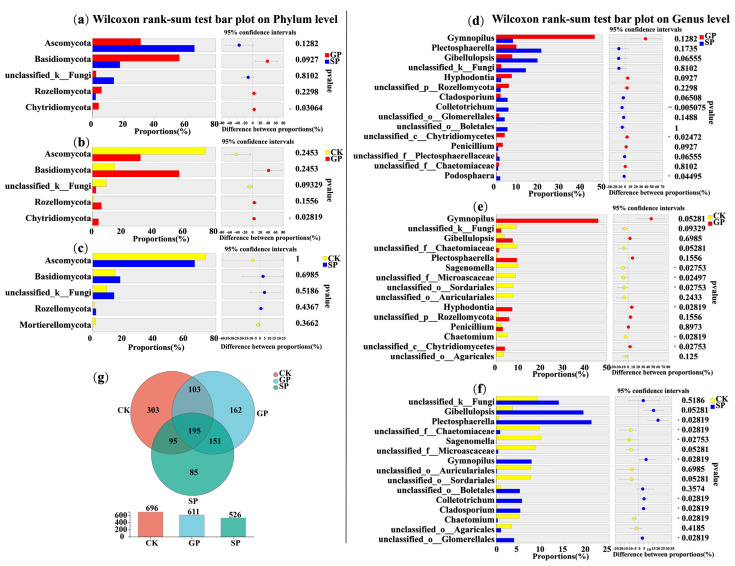
Significance analysis for soil fungi in the rhizospheres of giant-sized pumpkins (GPs), small-sized pumpkins (SPs), and background (CK) at the phylum (**a**–**c**) and genus (**d**–**f**) levels, as well as Venn diagram analysis at the OTU level (**g**).

Moreover, the Wilcoxon rank-sum test was also conducted to analyze the significant differences in the top five soil fungal phyla in terms of relative abundance percentage (Figure 8a). Only the abundance of Chytridiomycota was found to present a significant difference between the rhizospheres of the giant- (GP) and small-sized pumpkin (SP) varieties.

At the genus level, the proportions of *Colletotrichum*, *unclassified__c__Chytridiomycetes*, and *Podosphaera* in the rhizospheres of the giant- (GP) and small-sized pumpkin (SP) varieties presented significant differences. Moreover, the proportions of *unclassified_c__Chytridiomycetes* and *Colletotrichum* in the rhizospheres of the giant-sized pumpkin varieties (GP) were significantly higher than in those of the small-sized pumpkin varieties (SP). On the contrary, the proportion of *Podosphaera* in the rhizospheres of the small-sized pumpkin (SP) varieties was significantly higher than in those of the giant-sized pumpkin (GP) varieties (Wilcoxon rank-sum test, *, 0.01 < *p* ≤ 0.05; **, 0.001 < *p* ≤ 0.01; Figure 8d).

Furthermore, at the OTU level, the numbers of soil fungi in the rhizospheres of the giant-sized pumpkins, small-sized pumpkins, and CK were 611, 526, and 696, respectively. Meanwhile, the numbers of unique soil fungal OTUs in the rhizospheres of the giant-sized pumpkins, small-sized pumpkins, and CK were 162, 85, and 303, respectively. The above results indicate that various soil fungal compositions could be formed in the rhizospheres of different size pumpkin varieties. Furthermore, more unique soil fungi could be recruited in the rhizospheres of the giant-sized pumpkin varieties than in the small-sized pumpkin varieties (Figure 8g).

#### 2.4.2. LEfSe Analysis of the Fungal Communities at Phylum and Genus Levels

**Figure 9:**
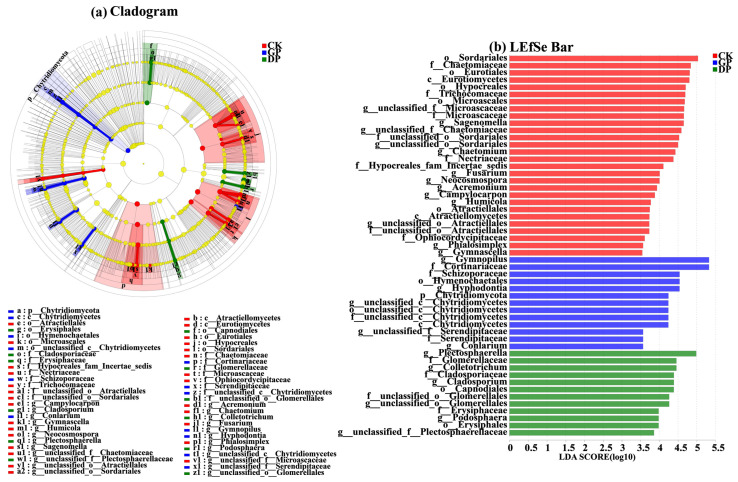
Cladogram showing the phylogenetic distribution of soil fungi in the rhizospheres of giant-sized pumpkins (GPs), small-sized pumpkins (SPs), and the background (CK). Indicator fungi with LDA scores of 3.5 or greater in microbial communities associated with soil from three treatments (LEfSe). Different color regions represent different constituents (blue: GP; green, SP; red: CK). Circles indicate phylogenetic level from phylum to genus. The diameter of each circle is proportional to the abundance of the group. Different prefixes indicate different levels (p: phylum; c: class; o: order; f: family; g: genus).

As shown in Figure 9, LEfSe analysis was performed on the fungal communities in the rhizospheres of the giant-sized pumpkins (GPs), small-sized pumpkins (SPs), and background (CK). A total 53 fungal species showed significant differences (*p* < 0.05, linear discriminant analysis [LDA] score > 3.5). Among them, at the phylum level, Chytridiomycota was significantly enriched in the rhizospheres of the giant-sized pumpkin (GP) varieties. However, there was no fungal phylum significantly enriched in the rhizospheres of the small-sized pumpkin varieties (SP). At the genus level, *Gymnopilus*, *Hyphodontia*, *unclassified_c__Chytridiomycetes*, *unclassified_f__Serendipitaceae*, and *Conlarium* were significantly enriched in the rhizospheres of the giant-sized pumpkin (GP) varieties. In contrast, *Plectosphaerella*, *Colletotrichum*, *Cladosporium*, *unclassified_o__Glomerellales*, *Podosphaera*, and *unclassified_f__Plectosphaerellaceae* were significantly enriched in the rhizospheres of the small-sized pumpkin (SP) varieties.

#### 2.4.3. Functional Predictions of Soil Fungal Community Structures

**Figure 10:**
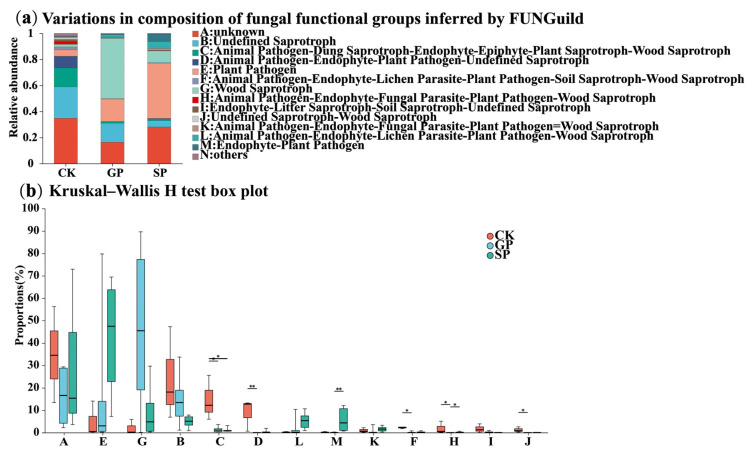
Relative abundance of FUNGuild-inferred functions (**a**) and Kruskal–Wallis rank-sum test (**b**) of soil fungi in the rhizospheres of giant-sized pumpkins (GPs), small-sized pumpkins (SPs), and background (CK). * indicates 0.01 < *p* < 0.05, ** indicates 0 < *p* < 0.01.

The Fungi Functional Guild (FUNGuild) function prediction method was used to predict the fungal functions in the rhizosphere of the differently sized pumpkin varieties. The results indicated that wood saprotroph, plant pathogen, animal pathogen-dung saprotroph-endophyte-epiphyte-plant saprotroph-wood saprotroph, animal pathogen-endophyte-lichen parasite-plant pathogen-wood saprotroph, and undefined saprotroph were the functional components with the top five relative abundances. In comparison with the small-sized pumpkin (SP) varieties, wood saprotroph and undefined saprotroph were significantly increased and Plant Pathogen was significantly decreased in the rhizospheres of the giant-sized pumpkin (GP) varieties (Figure 10).

## 3. Discussion

Previous studies have confirmed that soil micro-organisms in rhizospheres are closely related to plant growth and development. Rhizospheric micro-organisms can not only produce plant hormones and promote soil nutrient cycling but can also decompose root secretions through metabolic activities and affect plant growth and development [22]. Furthermore, endogenous and exogenous plant hormones have been confirmed to be important signaling substances in regulating plant fruit growth and development [23], and many exogenous plant hormones (e.g., auxin, gibberellin, cytokinin, abolic acid, and ethylene) can be derived from the soil microbiota in the rhizosphere [24], which could further affect plant growth [25,26]. Among them, auxin, gibberellin, and cytokinin promote cell division and cell growth during fruit development [27,28], facilitating the expansion of the fruit. On the contrary, abscisic acid and ethylene have opposite functions [29]. Additionally, soil micro-organisms in rhizospheres also play irreplaceable roles in the transformation of organic or inorganic substances in the soil, thus maintaining a healthy soil environment [30].

The phyla Firmicutes, Proteobacteria, and Actinobacteriota were found to be enriched in the rhizospheres of small-sized pumpkin (SP) varieties. Previous studies have reported that plant hormones, such as auxin and gibberellin, could be produced by Gemmatimonadota [31]. Furthermore, phosphate-dissolving compounds and nitrogen fixation activities can be carried out by Gemmatimonadota [32]. We found that the bacterial phylum Gemmatimonadota was significantly enriched in the rhizospheres of giant-sized pumpkin (GP) varieties when compared with the rhizospheres of small-sized pumpkin (SP) varieties. Moreover, Actinobacteriota has been significantly positively correlated with abscisic acid [25], and we also found that Actinobacteriota were significantly enriched in the rhizospheres of small-sized pumpkin (SP) varieties. Meanwhile, auxin and gibberellin have been reported to potentially be derived from *Streptomyces* [33,34], and we also found higher ratios of *Streptomyces* in the rhizospheres of giant-sized pumpkin (GP) varieties than in the rhizospheres of small-sized pumpkin (SP) varieties. Therefore, according to the enrichment and functions of the soil bacterial phyla and genera in the assessed rhizospheres, it could be speculated that there were higher contents of auxin and gibberellin, as well as a lower content of abscisic acid, in the rhizospheres of giant-sized pumpkin (GP) varieties than in those of small-sized pumpkin (SP) varieties.

Additionally, soil organic matter can be decomposed into inorganic minerals by Chytridiomycota, which could accelerate the nitrogen and phosphorus cycles in soil, improving their absorption by plants [35,36]. Meanwhile, cellulolytic enzymes may also be derived from Basidiomycota and *Gymnopilus*, thus promoting soil organic matter cycling [37,38]. Moreover, hydrolases and phytohormones can be produced by Cladosporium [39]. We found not only that Chytridiomycota was the unique dominant soil fungal phylum but also that the abundances of Basidiomycota and *Gymnopilus* were significantly increased in the rhizospheres of giant-sized pumpkin (GP) varieties compared with in those of small-sized pumpkin (SP) varieties. These results suggest that the circulation of soil organic matter could be accelerated in the rhizospheres of giant-sized pumpkin (GP) varieties when compared with in the rhizospheres of small-sized pumpkin (SP) varieties.

Furthermore, in comparison with small-sized pumpkin (SP) varieties, soil microbial functions—such as translation, ribosomal structure and biogenesis, nucleotide transport and metabolism, defense mechanisms, replication, wood saprotroph, and undefined saprotroph—were all higher in the rhizospheres of giant-sized pumpkin (GP) varieties.

## 4. Conclusions

The soil microbial composition in the rhizosphere was found to be differently shaped by giant- (GP) and small-sized pumpkin (SP) varieties. In particular, *norank_f__norank_o__Elsterales,* and *Ellin6067* were the unique dominant soil bacterial genera in the rhizospheres of giant-sized pumpkin varieties. By contrast, *Allorhizobium-Neorhizobium-Pararhizobium-Rhizobium*, *Arthrobacter*, *norank_f__Roseiflexaceae*, *Burkholderia-Caballeronia-Parab urkholderia*, and *Nocardioides* were the unique dominant soil bacterial genera in the rhizospheres of small-sized pumpkin varieties. 

Furthermore, *unclassified_p__Rozellomycota* and *unclassified_c__Chytridiomycetes* were the unique dominant soil fungal genera in the rhizospheres of giant-sized pumpkin (GP) varieties, while *Podosphaera*, *Colletotrichum*, and *unclassified_f__Plectosphaerellaceae* were the unique dominant soil fungal genera in the rhizospheres of small-sized pumpkin (GP) varieties. Among these, soil bacterial genera such as *norank_f__norank_o__Elsterale*, *Ellin6067*, *norank_f__67-14*, and *Chujaibacter* and fungal genera such as *unclassified__p__Rozellomycota*, *unclassified__c__Chytridiomycetes*, *Penicillium*, and *unclassified__f__Chaetomiaceae* can be speculated as potential soil functional micro-organisms associated with improved pumpkin size.

## 5. Materials and Methods

### 5.1. Field Site Description

The experiment was conducted at the experimental vegetable base (108°17′25″ E, 22°51′02″ N) of the Agricultural College, Guangxi University. The soil type was red loam with pH 5.12, and the contents of organic matter, total nitrogen, phosphorus, and potassium were 8.17 g kg^−1^, 0.65 g·kg^−1^, 0.67 g·kg^−1^, and 7.52 g·kg^−1^, respectively. Meanwhile, the contents of available nitrogen, phosphorus, and potassium were 15.27 mg·kg^−1^, 0.77 mg·kg^−1^, and 81.8 mg·kg^−1^, respectively.

### 5.2. Experimental Design

**Figure 11:**
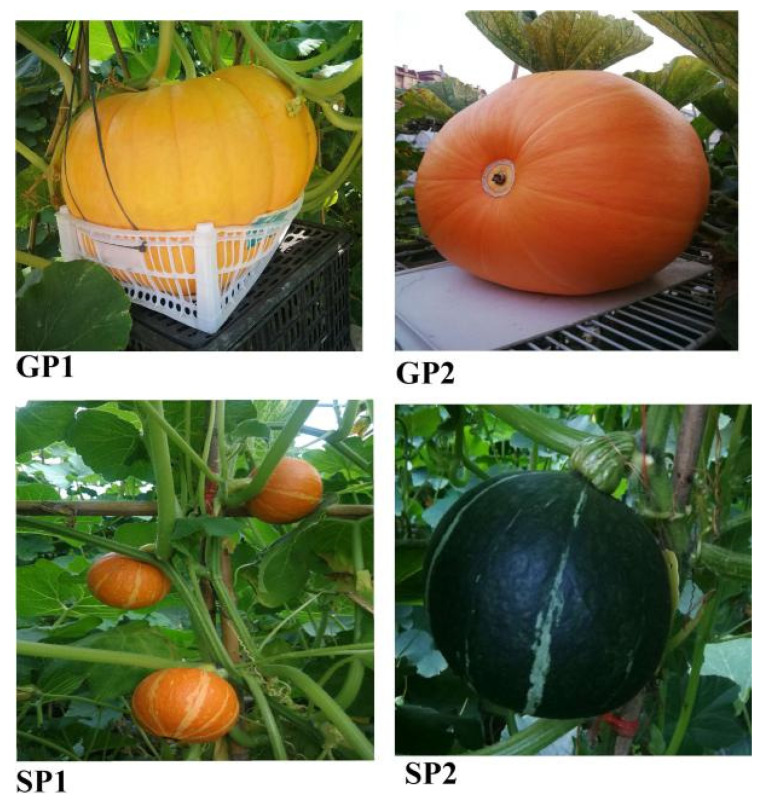
Giant- (GP) and small-sized (SP) pumpkin varieties.

The trial was conducted from February to August 2021 under identical management conditions. The giant-sized pumpkin varieties “JUNNAN1HAO” (GP1) and “JUNNAN2HAO” (GP2) and small-sized pumpkin varieties “JINXING” (SP1) and Gui Li 2 (SP2) were used in this study (Figure 11).

### 5.3. Soil Sample Collection

Rhizospheric soil samples from each variety were collected on 15 June 2021, during the ripening period of the pumpkin fruit, using the shaking-off method. A shovel was first disinfected with 75% ethanol and then used to loosen soil in a 30 cm radius around the plants, following which the whole plants were pulled out [40]. After the bulk soil was shaken off, rhizospheric soil samples were carefully collected from the roots, put into sterile plastic bags, and transferred to the laboratory immediately if possible. Soil samples were sieved through a 2 mm stainless steel mesh and stored in a refrigerator at 4 °C for immediate analysis or at −80 °C for later use. Meanwhile, soil samples from the same field without pumpkin growing were also used as background samples (CK).

### 5.4. Soil Bacterial DNA Extraction and Illumina Sequencing

Total DNA extraction, PCR amplification, and sequence determination were performed sequentially following previously defined protocols [41]. PCR amplification of the V3-V4 region was performed using the bacterial primers 338F (5′-ACTCCTACGGGAGGCAGCAG-3′) and 806R (5′-GGACTACHVGGGTWTCTAAT-3′), whereas PCR amplification of the fungal ITS1 region was performed using ITS1F (5′-CTTGGTCATTTA-GAGGAAGTAA-3′) and ITS2R (5′-GCTGCGTTCTTCATCGATGC-3′) primers. The original reads were stored in the NCBI Sequence ReadArchive (SRA) database (Accession Number: SRP366137). The 300 bp reads were truncated using Quantitative Insights into Microbial Ecology (QIIME; version 1.17; average quality score < 20 over a 50 bp sliding window). UPARSE (version 7.1, https://drive5.com/uparse/, accessed on 15 August 2023) was used to cluster operational taxonomic units (OTUs) with a 97% similarity cut-off, and chimeric sequences were recognized and eliminated. The RDP Classifier (https://rdp.cme.msu.edu/, accessed on 17 August 2023) was used to analyze the taxonomy of each OTU representative sequence to the 16S rRNA database with a confidence threshold of 0.7. Mothur (version v.1.30.2; https://mothur.org/wiki/calculators/, accessed on 20 August 2023) was used to calculate the alpha diversities of the microbial communities.

### 5.5. Statistical Analysis

Excel 2019 and IBM SPSS Statistics 26 were used to analyze the experimental data, and the results are shown as the means with their standard deviations (mean ± SD). Wilcoxon rank-sum test was conducted to analyze the significance of differences in the statistical analyses (*p* < 0.05).

The alpha diversities of the rhizosphere bacterial and fungal communities were calculated using Mothur (version v.1.30.2, https://mothur.org/wiki/calculators/, accessed on 15 August 2023). Quantitative Insights into Microbial Ecology (QIIME; version 1.17) was used to truncate the 300 bp reads (average quality score < 20 over a 50 bp sliding window) [42]. OUPARSE (version 7.1, http://drive5.com/uparse/, accessed on 15 August 2023) was used to cluster operational taxonomic units (OTUs) with a 97% similarity cutoff, and chimeric sequences were found and eliminated [43]. The taxonomy of each OTU representative sequence was analyzed using the RDP Classifier (http://rdp.cme.msu.edu/, accessed on 15 August 2023) against the 16S and ITS rRNA databases, with a confidence threshold of 0.7 [40]. A principal component analysis (PCA) based on the unweighted UniFrac and principal coordinate analysis (PCoA) was performed to evaluate the similarity of the rhizosphere microbial communities, and the R language (version 3.3.1) was used for statistical analysis and graphical representation. The visual circles of the bacterial and fungi communities were described using Circos-0.67–7 (http://circos.ca/, accessed on 20 August 2023). OTU tables with a 97% similarity level were selected for microbial community composition and Venn diagram analysis, and the R language (version 3.3.1) was used for statistics and graphical representation. A linear discriminant analysis (LDA) was performed using LEfSe (http://huttenhower.sph.harvard.edu/galaxy/root?tool_id=lefse_upload, accessed on 15 August 2023) on samples according to different grouping conditions based on taxonomic composition in order to identify clusters that had a significant differential impact on sample delineation. Meanwhile, PICRUSt was used to estimate the functional components of bacterial communities using the Kyoto Encyclopedia of Genes and Genomes (KEGG) dataset. Functional predictions of the fungal communities were performed using the Fungi Functional (FUN) Guild tool [44]. Online data analysis was performed using the Majorbio Cloud Platform (www.majorbio.com, accessed on 19 August 2023) from Majorbio Bio-Pharm Technology Co., Ltd. (Shanghai, China).

## Figures and Tables

**Table 1:** Soil bacterial diversities in the rhizospheres of giant- and small-sized pumpkin varieties.

Varieties	Shannon	Simpson	Ace	Chao	Coverage
CK	5.97 ± 0.87 a	0.0112 ± 0.0128 a	2098.23 ± 713.91 a	2068.36 ± 671.75 a	0.98
GP1	6.00 ± 0.19 a	0.0063 ± 0.0012 a	2536.10 ± 165.58 a	2377.70 ± 194.07 a	0.97
GP2	6.07 ± 0.28 a	0.0061 ± 0.0019 a	2394.43 ± 251.52 a	2367.29 ± 238.41 a	0.97
SP1	6.21 ± 0.21 a	0.0053 ± 0.0011 a	2513.65 ± 183.76 a	2490.90 ± 218.47 a	0.97
SP2	6.13 ± 0.09 a	0.0064 ± 0.0011 a	2464.29 ± 125.13 a	2470.67 ± 131.42 a	0.97

Note that all statistics are presented as the mean ± SD (standard deviation). Significant variations between treatments at *p* < 0.05 are indicated by different letters in the same column.

**Table 2:** Soil fungal diversities in the rhizospheres of giant- and small-sized pumpkin varieties.

Varieties	Shannon	Simpson	Ace	Chao	Coverage
CK	3.21 ± 0.68 a	0.09 ± 0.02 a	382.64 ± 237.19 a	379.97 ± 236.57 a	0.99
GP1	1.66 ± 0.97 a	0.43 ± 0.33 a	360.87 ± 68.63 a	319.27 ± 86.93 a	0.99
GP2	2.07 ± 1.52 a	0.36 ± 0.35 a	351.20 ± 116.11 a	318.44 ± 140.97 a	0.99
SP1	2.17 ± 0.21 a	0.22 ± 0.06 a	331.65 ± 35.90 a	315.64 ± 16.24 a	0.99
SP2	2.31 ± 0.36 a	0.20 ± 0.05 a	314.96 ± 82.23 a	311.79 ± 79.36 a	0.99

Note that all statistics are presented as the mean ± SD (standard deviation). Significant variations between treatments at *p* < 0.05 are indicated by different letters in the same column.

## Data Availability

Raw data for bacterial and fungal bacterial sequences were deposited in the NCBI Sequence Read Archive (SRA) database under accession numbers PRJNA1003782 and PRJNA1003827, respectively.
